# Television Viewing and Cognitive Dysfunction of Korean Older Adults

**DOI:** 10.3390/healthcare8040547

**Published:** 2020-12-10

**Authors:** Mi Sook Jung, Eunyoung Chung

**Affiliations:** 1 College of Nursing, Chungnam National University, Daejeon 35015, Korea; msjung@cnu.ac.kr; 2 Daejeon Metropolitan City Public Health Policy Institute, Daejeon 35015, Korea

**Keywords:** television viewing, sedentary behavior, leisure activities, cognitive dysfunction, aged

## Abstract

This study examined the association between television (TV) viewing and cognitive dysfunction in elderly Koreans. Among participants of the 2014 National Survey of Older Koreans, 9644 were considered in this study. To better identify the association between two factors, propensity score (PS) matching with exact method was used. Finally, 168 viewers and non-viewers each were selected based on estimated PS on key variables and eliminating double matches. Multivariate logistic regression analysis was performed when controlling for possible covariates. Viewers were more likely to have cognitive dysfunction than non-viewers, with significant differences in most covariates. After correcting confounding effects of these covariates with PS matching, TV viewing was found to be a significant risk factor of cognitive dysfunction, along with absence of diagnosed hypertension and non-participation in physical leisure activities. TV viewing might be associated with increased risk of cognitive dysfunction in later life. Appropriate education and strategies to minimize TV viewing among older adults should be established to contribute to attenuating cognitive aging. More interventional studies can help older adults, caregivers, and healthcare professionals explore the cognitively beneficial alternatives to TV use considering the impact of socioeconomic factors of selecting TV viewing as a preferred leisure activity.

## 1. Introduction

Cognitive dysfunction is the leading cause of concern regarding the aging of older adults because age is a major non-modifiable risk factor for the development of dementia [1]. In 2017, there were an estimated 50 million people living with dementia worldwide and this number is likely to rise to about 152 million people worldwide by 2050 [2]. According to a national statistic from South Korea, the prevalence rate of dementia is rapidly increasing because the pace of growth of the aging population is unprecedented with a fast transition from an aging society (7% in 2000) to an aged one (14% in 2017) in only 17 years [3]. Therefore, healthy cognitive aging and dementia prevention methods have become priorities for maintaining high levels of functional abilities with an emphasis on independent living.

Engaging in leisure activities has consistently been reported to be beneficial for late-life cognitive functioning [4,5]. Previous cohort studies have supported the theory that the onset of dementia can be delayed by both physical and cognitive leisure activities among community-dwelling older adults [6,7]. The effects of these leisure activities on cognitive function are associated with developing cognitive reserves in the brain and reducing modifiable risk factors of dementia such as cardiovascular diseases, thereby slowing age-related declines in cognitive functioning and protecting brain functioning from pathological damage [8].

Television (TV) viewing is the most common leisure activity among older adults in real-life settings because TV viewing is easily accessible and does not involve barriers to participation as some other leisure activities do [9]. The national statistics on Korea’s older population (those aged over 65 years old) revealed that in 2015, their average amount of spare time per day was over 7 h and they watched TV for approximately 4 h each day [10]. Similarly, the American Time Use survey in 2018 reported that individuals aged 65 years or older spent the most time watching TV compared to other age groups; those aged 65 to 74 years old averaged 4.34 h of TV viewing per day and those aged 75 years or older averaged 4.78 h of TV viewing per day [11]. Some studies have found that TV viewing is associated with a potentially high risk of cognitive dysfunction and therefore cannot substitute cognitively stimulating activities that have benefits for neurological functioning [12,13,14,15]. Although the mechanism underlying this relationship between more time spent watching TV and poorer cognitive performance is unknown, three hypothetical explanations include (1) potential cognitive stress created through “alert-passive interaction” while watching TV, (2) a reduction in the amount of time spent on physical activities that are beneficial for cognitive preservation, and (3) a behavioral tendency toward spending more time watching TV among individuals with cognitive dysfunction such as dementia [13,14,15,16].

Due to the combination of heavy TV viewing among older adults and its negative effect on late-life cognition, attention to this association is rapidly growing. To the best of our knowledge there may be a significant association between TV viewing during mid-life and cognitive decline and increased risk of dementia among older adults. However, no study has observed the effects of TV viewing on cognitive dysfunction within the context of daily life among the older population participating in various leisure activities with cognitive benefits. Therefore, this study aimed to examine the association between TV viewing and cognitive dysfunction among individuals aged 65 years or older by using a nationally representative sample. Propensity score (PS) matching was used to correct the selection of observables in estimates of the association analyzed in this study. This approach allowed us to better explain the effects of potential confounders and moreover, to easily generalize our results to the overall population.

## 2. Methods

### 2.1. Study Sample

Data were obtained from the National Survey of Older Koreans (NSOK), which is a nationally representative cross-sectional survey conducted by The Korean Institute for Health and Social Affairs (KIHASA). The KIHASA performed this survey every three years between 2008 and 2019 and each survey was comprised of slightly different questionnaires about living conditions, needs, and desires of older people residing in communities. We used the 2014 NSOK dataset because it was the latest survey including a variable regarding TV viewing along with other leisure activities, health, lifestyles, living arrangements and conditions, and quality of life. Among the original data of a total of 10,451 individuals in the 2014 NSOK, we used the data of 9644 participants who had provided details on cognitive performance, and key variables used for matching criteria were selected based on previous studies including engagement in leisure activities, social network and participation, health condition, and health-related behaviors [17,18]. Participants consisted of 1776 individuals who did not spend any time watching TV (non-viewers) and 7888 individuals who spent at least some leisure time watching TV (viewers). There were 405 participants (174 non-viewers and 231 viewers) who exactly matched the statistical adjustments for residual confounders. Finally, 336 participants (168 non-viewers and 168 viewers) were included after eliminating double matches. The Institutional Review Board of Chungnam National University approved this study (201711-SB-087-01).

### 2.2. Measures

This study assessed information regarding TV viewing as an independent variable, cognitive dysfunction as an outcome, and engagement in cognitive and physical leisure activities, social network and participation, health condition, and health-related behaviors as covariates.

#### 2.2.1. Cognitive Dysfunction

Scores on the Korean version of the Mini-Mental Status Examination for Dementia Screening (MMSE-DS), a national screening tool for cognitive dysfunction formally designed for use by the Ministry of Health and Welfare in Korea, was used to identify the presence of cognitive dysfunction [19]. The MMSE-DS contains 19 items on orientation, verbal memory, concentration, calculation, language, praxis, and visuospatial construction and the maximum total score is 30 points. In this study, cognitive dysfunction was screened with age-, education-, and sex-adjusted scores falling 1.5 standard deviations below that expected for age and education level of male and female Korean older adults, according to the national guideline covering diagnosing and managing dementia [20]. This standardized test has been confirmed as a reliable and valid instrument for screening cognitive dysfunction such as dementia [19]. Table 1 shows the suggested cutoff scores adjusting for age, education, and sex.

#### 2.2.2. Television Viewing

TV viewing was assessed using two questions on participants’ leisure activities: “Have you engaged in any leisure activities over the past one year?” and “Please rank the top three frequent leisure activities that you usually engage in.” If participants answered ‘yes’ to the first question, the second question was given to them. If participants named TV viewing as one of their top three leisure activities, they were considered “viewers”; if participants did not mark TV viewing as one of their three frequently engaged leisure activities, they were considered “non-viewers”.

#### 2.2.3. Covariates

Covariates were selected based on the cognitive reserve theory [17] and previous epidemiological studies on the predictors of developing dementia [18,21,22,23,24,25,26]. Engagement in cognitively beneficial leisure activities, social network and social participation, health condition (i.e., presence/absence of cardiovascular disease, depressive symptoms, or perception problems), health-related behaviors (i.e., current smoking or drinking practices and exercise), and sociodemographic factors (i.e., age, sex, years of education, and residential location) were assessed.

Leisure activities were assessed with the same questions used for TV viewing. All the activities that the participants named were classified into 24 activities and then categorized as cognitive or physical according to the criteria established in a previous study [27] and two systematic reviews [5,28]. Cognitive activities contained intellectually stimulating activities such as playing musical instruments, painting, playing card games, and reading. Physical activities ranged from unstructured to structured physical exercise (e.g., walking, jogging, dancing, playing tennis, and swimming). Individuals’ participation in each activity was coded on a binary form that indicated whether they participated in such activities with cognitive benefits.

Three dichotomous variables regarding the participants’ social networks were evaluated via the frequency of face-to-face and other types of contact based on previous studies [29,30]. Participants were asked how often they met or contacted (via telephone, mobile phone text, email, letter etc.) their children, siblings/relatives, and friends/neighbors over the past year. Respondents rated their answers on eight-point Likert scales from 0 “hardly any contact/visiting” to 7 “contact/visit more than four times per week”. “Hardly any contact/visiting” was considered to represent the absence of a social network; all other responses were considered to represent the presence of a social network.

Social participation was assessed using three dichotomous variables describing participants’ involvement in the following activities based on the previous literature [31,32]: currently engaging in paid work, participating in continuing education, and doing volunteer work. These questions were answered with “yes” or “no”.

The health condition was assessed by using questions regarding cardiovascular diseases, depressive symptoms, and perceptual problems such as hearing and visual difficulties that were selected as risk factors of dementia evidenced by several previous studies [23,24,26]. Among the questions about current health problems, two questions about hypertension and diabetes were used to assess the presence of cardiovascular diseases. Participants’ answers were divided into the categories of “yes” and “no”. Depressive symptoms were assessed using the Korean version of the Geriatric Depression Scale-Short Form (SGDS-K). This measure was developed to identify the presence of depression in older adults in community and clinical care settings [33,34]. It consists of 15 items with “yes” or “no” answers. Total scores can range from 0 to 15 and a score above 8 indicates the presence of depressive symptoms in Korean older adults [35]. SGDS-K was confirmed as a reliable and valid instrument in a study of older individuals with and without clinical depression [34,36]. The Kuder–Richardson Formula 20 coefficient for all items in this scale was 0.904, indicating high reliability. Perceptual problems including hearing and visual difficulties were evaluated with two questions: “Do you wear a hearing aid or glasses?” and “Do you feel uncomfortable regardless of wearing hearing aid or glasses because of perceptual problems in your daily life, such as when you watch TV or talk to someone on the phone?”. The response options for the latter question were “comfortable”, “uncomfortable”, and “very uncomfortable”, and the two negative responses were combined for dichotomizing these variables.

Health-related behaviors were assessed using questions about current smoking and drinking practices [21,25]. Data on current smoking were obtained from the question: “Do you smoke cigarettes now?” with dichotomous response options, and data on current drinking were obtained from a question about the frequency of alcoholic beverage consumption over the past year. If participants chose “I have not drunk at all in one year”, their answers were recoded as “no”.

### 2.3. Stastistical Analysis

Propensity Score (PS) matching with the exact method was used to define a comparison group from the group of older adults categorized as leisure time TV viewers in order to compare the group of individuals who were not involved in TV viewing as their leisure activities. This matching method consists of matching each experimental unit with a control unit that has exactly the same values on each covariate to ensure that individuals are paired on key variables of interest [37]. Our matching criteria for analysis were age (years), sex (male/female), years of education, residential location (rural/urban), cardiovascular diseases (hypertension and diabetes), presence of depressive symptoms (yes/no), having perceptual problems (yes/no), current cigarette smoking (yes/no), current alcohol consumption (yes/no), participation in cognitive leisure activities (yes/no), participation in physical leisure activities (yes/no), contact with/visiting children (yes/no), contact with/visiting relatives (yes/no), contact with/visiting friends (yes/no), current employment status (employed/unemployed), engagement in continuing education (yes/no), and participation in volunteer work (yes/no). All procedures were conducted with the statistical program R (version 3.5.2) and a randomization tool available on the website www.randomizer.org [38] was used to randomly select final matched pairs while eliminating double matches.

Sociodemographic characteristics, engagement in leisure activities, social networks and participation, health condition, and health-related behaviors were evaluated using frequency and descriptive analyses. The balance between cases and controls before and after matching were checked by independent t tests and chi-square analyses. Multivariate logistic regression analyses were performed to the dataset before PS matching and to the exact-matched dataset after PS matching in order to examine the relationship between TV viewing and cognitive dysfunction before and after controlling for the residual effects of possible covariates. In this model, cognitive dysfunction was included as an outcome variable, TV viewing as an independent variable, and the selected 19 variables used for matching criteria were included as covariates. SPSS Version 22.0 statistical software (IBM Corp., Armonk, NY, USA) was used in all statistical analyses.

## 3. Results

### 3.1. Characteristics of Study Groups

Sociodemographic characteristics, leisure activities, social networks and participation, health condition, and health-related behaviors of the TV viewer and comparison groups before and after PS matching are presented in Table 2. These results showed that 81.62% of all participants were leisure time TV viewers (*n* = 7888) and the TV viewer group differed from the comparison group significantly regarding almost all characteristics before matching. Specifically, the viewer group was older, less educated, consisted of more women, resided more often in rural areas, had higher percentages of having diabetes, had more depressed moods, experienced more visual or hearing problems, and were more likely to currently smoke than the non-viewer group. Moreover, individuals in the viewer group reported smaller social networks, less participation in social activities, and less engagement in cognitively beneficial leisure activities (e.g., cognitive and physical activities) than those in the comparison group. However, there were no significant differences in these variables between the two groups after the exact matching.

### 3.2. Cognitive Functioning of Study Groups

As presented in Table 2, the viewer group had lower scores on MMSE-DS than that in the non-viewer group (t = 16.07, *p* < 0.001, before PS matching; t = 2.09, *p* = 0.037, after PS matching). The occurrence of cognitive dysfunction was defined using a norm-referred cutoff point for each individual (20). The viewer group showed significantly higher proportions of individuals having MMSE-DS scores lower than cutoff points than the non-viewer group (χ^2^ = 52.71, *p* < 0.001, before PS matching; χ^2^ = 6.24, *p* = 0.017, after PS matching).

### 3.3. Multivariable Analysis of Cognitive Dysfunction

The results of the multivariate logistic regression before and after PS matching are presented in Table 3. With the original data before matching, the odds ratios (OR) and 95% confidence intervals (CI) of cognitive impairment were calculated. The OR was 1.27 (95% CI = 1.12–1.45) for individuals having a membership of the TV viewer group, compared with those in the non-viewer group. One unit change in age and education was associated with 1.02 (95% CI = 1.01–1.03) and 1.03 (95% CI = 1.02–1.04) increase in the OR in this model, respectively. As compared with individuals residing in the urban areas, the OR was 1.49 (95% CI = 1.34–1.65) for those living in rural areas. The OR for individuals having hypertension diagnosed through medical examination in a hospital was 1.22 (95% CI = 1.11–1.33) compared with those without hypertension while the OR for individuals with diabetes was 0.86 (95% CI = 0.77–0.96) compared with those without diabetes. The OR was 1.31 (95% CI = 1.18–1.44) for depressed individuals compared with those having no depressive symptoms. The OR was 1.17 (95% CI = 1.06–1.28) for individuals with visual difficulties compared with those without visual problems, while the OR was 1.33 (95% CI = 1.20–1.48) for individuals with hearing difficulties compared with those without hearing difficulties. Individuals who did not engage in cognitive activities had 20% higher odds of cognitive dysfunction (OR = 1.20, 95% CI = 1.07–1.35) than those involved in cognitive activities, while the association was not statistically significant for those categorized by being involved in physical activities (OR = 1.01, 95% CI = 0.91–1.12). The OR increased significantly by 45% (OR = 1.45, 95% CI = 1.27–1.66) and 53% (OR = 1.53, 95% CI = 1.25–1.86) for individuals who did not come into contact with their relatives or friends compared with those who contacted their social groups, respectively. Individuals who did not engage in lifelong education or volunteer work had 59% (OR = 1.59, 95% CI = 1.37–1.84) and 41% (OR = 1.41, 95% CI = 1.10–1.81) higher odds of cognitive dysfunction than those who engaged in education and participated in volunteer work.

The results of the multivariable logistic regression with data after PS matching revealed that three variables remained as factors associated with cognitive dysfunction: having a membership of the TV viewing group, hypertension, and involvement in physical activities. The OR (OR= 1.94, 95% CI = 1.18–3.21) for individuals in the viewer group approximately doubled compared with that calculated before PS matching. The OR still significantly increased for individuals without hypertension compared with those with hypertension (OR = 2.14, 95% CI = 1.18–3.88). Participation in physical activities was found as a new factor associated with the outcome variable after controlling the residual effects of other covariates with PS matching (OR = 2.11, 95% CI = 1.05–4.22).

## 4. Discussion

This study was the first report of identifying the association between TV viewing and cognitive dysfunction by using nationally representative data of Korean adults aged over 65 years old. We found that the leisure time TV viewers were more likely to have cognitive dysfunction than the PS-matched non-viewers. In addition to the engagement in TV viewing as a leisure activity, the absence of diagnosed hypertension and the lack of participation in physical leisure activities were significantly associated with the presence of cognitive dysfunction. However, other risk factors that were significantly associated with cognitive dysfunction before PS matching were not found to be significantly associated with cognitive dysfunction after PS matching. Our findings suggest that TV viewing, the most common leisure activity of older adults, may not support older adults in maintaining cognitive functioning or reducing the risk of dementia.

Previous studies examining the relationship between TV viewing and late-life cognition have reported mixed results [15,39,40]. In several cross-sectional and longitudinal studies, TV viewing has been categorized as a cognitive, mental, intellectual, passive, and recreational activity, indicating it may be one of the leisure activities that has potentially preventive effects against cognitive dysfunction in old age. These studies measured TV viewing and other cognitively stimulating leisure activities together as one composite score, which showed a positive correlation with late-life cognition [7,39,40,41]. However, this finding only showed the positive association of all measured activities overall, not the association of TV viewing with cognitive function individually. In contrast, several studies have reported a negative association between TV viewing and cognitive function. For example, a population-based study indicated that excessive TV viewing was associated with worse executive functioning among 2579 older adults in both longitudinal and cross-sectional analyses [13]. A similar result was obtained in a case-control study examining the effects of mid-life TV viewing behavior on the occurrence of late-life cognitive dysfunction among 135 individuals with Alzheimer’s disease and 331 healthy controls. Individuals with Alzheimer’s disease were likely to spend 25.51% of their leisure time in middle adulthood (40–59 years old) on watching TV while healthy controls spent 19.04% of their leisure time during the same life stage. The negative effect of daily TV viewing on late-life cognitive function persisted even when controlling for participation in cognitively-beneficial activities and sociodemographic characteristics, with a 1.3 times greater risk of belonging to the Alzheimer’s group if an individual’s TV viewing time increases by 1 h [42]. Interestingly, this suggested the necessity of examining the effects of the contents of frequently watched TV programs on late-life cognition. In a study with 289 older women, talk shows and soap operas which were their favorite TV programs were commonly associated with delayed response times for correctly performing the Trail Making Test, fewer correct answers on the Hopkins Verbal Learning Test-Revised, and lower scores on the MMSE. When controlling for possible covariates such as sociodemographic factors, cardiovascular diseases, and depressive symptoms, some significant relationship still remained between types of favorite television programs watched and cognitive function [43].

There are three hypothetical explanations about the association between engaging in TV viewing behavior and cognitive functioning. First, fast-paced changes in sensory stimuli such as images, sounds, and action on a TV screen can direct viewers’ attention to such visual and auditory information but may fail to properly stimulate relevant mental activity, which is required for the elaborate integration with higher-level cognitive processing in the brain [15]. This alert-passive interaction is especially prominent in TV viewing when compared to other screen-based activities such as Internet use that show enhanced multisensory integration that leads to a reduction of the risk for dementia [44]. A neuroimaging study showed that individuals’ ability to control their focus of attention tended to decline with age and that neural responses in older adults with less attentional control may be less effective in ignoring internally- and externally-generated distraction during memory and comprehension tasks as cognitive demand increased [45]. These findings suggest that attentional control reduction with age and the alert-passive interaction between perceptual stimuli generated on screen and TV viewers may be associated with non-beneficial effects on cognitive function. Second, another explanation is that spending more time watching TV can deprive them of time to engage in other cognitive or physical activities that have been known to be cognitively beneficial in everyday life [46]. In the scientific literature, it is frequently reported that more hours spent per week on watching TV was associated with the presence of cognitive dysfunction defined using the cutoff score of the MMSE and less hours spent in cognitive or intellectual leisure activities such as playing board games, reading, and writing [42,47]. Our study showed similar results in that leisure time TV viewers were less likely to engage in cognitive and physical activities than non-viewers. Third, individuals with cognitive dysfunction were likely to show the behavioral tendency of spending more time watching TV than those with no cognitive dysfunction [13,43]. This behavioral tendency might be related to diminished cognitive abilities and exaggerated dependency on the environment for behavioral cues. In a study with individuals with frontotemporal dementia and Alzheimer’s disease, 39.6% and 57.5% of the patients showed an increase in TV viewing time, respectively, which was evident both before and after disease onset. Excessive TV viewing, defined as watching TV for more than 12 h a day, was more prevalent in patients with frontotemporal dementia than those with Alzheimer’s disease [48]. Moreover, patients with more than three signs of environmental dependency spent more hours watching TV per day than those with three or fewer signs [48]. However, the mechanisms underlying this behavioral tendency remain unclear due to absence of longitudinal assessment during the transition period before and after dementia diagnosis. Additional research is needed to clarify the change in TV viewing behavior and its impact on changes in late-life cognitive function and onset of dysfunction to directly test this hypothesis.

Unexpectedly, individuals without high blood pressure had higher odds of cognitive dysfunction than those with hypertension in our study. This finding was consistent with previous studies showing that elevated late-life blood pressure was associated with a reduced risk of developing dementia [49,50]. A recent review reported that the relationship between blood pressure and occurrence of dementia is age-dependent, suggesting that mid-life hypertension increases the risk of dementia, though late-life hypertension does not seem to show the same effect [51]. A population-based study with cognitively healthy 2356 older adults aged from 65 to over 85 years reported that the hazard ratios of demonstrating dementia symptoms occurring in the hypertensive group compared to the non-hypertensive group tended to decrease from 1.38 in the group aged between 65 and 74 years, 0.94 in those aged between 75 and 84 years, and to 0.70 in those aged over 85 years [49]. Additionally, there was a positive relationship between higher systolic blood pressure and occurrence of dementia in the youngest group (aged between 65 and 74 years), while no significant relationships between these two factors were found in other 2 groups aged over 75 years, reflecting a possible protective effect of elevated blood pressure in later life [49]. The mechanisms underlying the age-dependent association of hypertension are still not clear. Furthermore, our study added evidence on this cross-sectional association. Thus, future studies could identify the causal relationship using a longitudinal design.

Participation in physical activities was significantly associated with cognitive dysfunction with an increased odds ratio for individuals who were not physically active compared with those who were. The positive effect of physical leisure activities on late-life cognition found in this study is well established in several previous studies [4,52]. A systematic meta-analysis of prospective studies with a follow-up period of 1–12 years revealed that older adults who were physically active at baseline had a reduced risk of developing cognitive decline [4]. Moreover, neuroimaging findings support the neuroprotective effect of physical activities by showing that performing physical activities increased the volume of gray matter in the brain, and contributed to generate several factors related to strengthening synapses between neurons or facilitating neuro-genesis, such as brain-derived neurotrophic factor, insulin-like growth factor, and vascular endothelial growth factor [53,54].

There are some limitations that need to be cautiously interpreted. The nature of a cross-sectional study may be subject to reverse causality. For example, older adults with cognitive dysfunction were more likely to choose TV viewing for their leisure time than cognitively healthy adults due to the lowered cognitive functioning. Moreover, the lack of detailed information such as types of TV programs and the amount of TV viewing time would prevent us from capturing the possible multifaceted relationship of TV viewing and late-life cognition, given that the previous studies have demonstrated significant association with TV viewing [15,43]. Furthermore, the original questions about leisure activities of the dataset used in this study was designed to answer relying on participants’ memory. Considering cognitive characteristics of older people, the questions need to be revised to reduce the demand of individuals’ abilities to retrieve information. There is another methodological limitation regarding the outcome variable in this study. Cognitive dysfunction was defined with norm-referred cutoff scores of the MMSE-DS in this study. Future research should include advanced medical measures such as neuropsychological test battery or neuroimaging techniques to define cognitive dysfunction in older adults. Finally, the participants excluded from analysis because of unavailability of key variables would limit the representativeness of data. Nevertheless, PS matching analysis used in this study provided an unbiased estimate of the association of TV viewing behavior with cognitive dysfunction with an exactly matched sample, supporting that leisure time TV use, even in later life, was inversely associated with late-life cognition independent of other beneficial leisure activities, sociodemographics, and health-related factors.

In conclusion, analysis with an exactly matched sample in the present study highlighted the inverse association of leisure time TV viewing with late-life cognition by controlling for residual effects of various covariates on late-life cognitive functioning evidenced by previous studies. Therefore, a suitable strategy to keep older adults away from TV should be established to attenuate cognitive decline in older adults. Moreover, prospective studies are needed to explore the causal relationship between TV viewing and cognitive functioning with more elaborated data indicating the frequency or the amount of time used for watching TV, and more interventional studies should explore the cognitively-beneficial alternatives of TV use, considering the impact of socioeconomic factors linked to the leisure time of TV use.

## Figures and Tables

**Table 1:** Norm-referenced cutoff scores of Mini-Mental Status Examination for Dementia Screening (MMSE-DS).

Age	Sex	Education (Year)			
		0–3	4–6	7–12	≥13
60–69	Male	20	24	25	26
	Female	19	23	25	26
70–74	Male	20	23	25	26
	Female	18	21	25	26
75–79	Male	20	22	25	25
	Female	17	21	24	26
≥80	Male	18	22	24	25
	Female	16	20	24	26

**Table 2:** Descriptive statistics of Korean older adults by TV viewing.

Variables		Before Matching			After Matching		
		TV Viewing		χ^2^ or t	TV Viewing		χ^2^ or t
		Yes (*n* = 7888)	No (*n* = 1776)		Yes (*n* = 168)	No (*n* = 168)	
Cognitive performance		23.34 ± 4.72	25.41 ± 4.35	16.97 ***	24.22 ± 4.65	25.26 ± 4.38	2.09 *
Age, Mean ± SD, year		74.08 ± 6.50	72.50 ± 6.14	−9.29 ***	71.2 ± 4.84	71.2 ± 4.84	0.00
Sex	Male	3203 (40.6)	852 (48.0)	32.35 ***	57 (33.9)	57 (33.9)	0.00
	Female	4686 (59.4)	924 (52.0)		111 (66.1)	111 (66.1)	
Education, Mean ± SD, year		6.06 ± 4.67	8.88 ± 5.12	22.55 ***	7.82 ± 4.64	7.82 ± 4.64	0.00
Residential location	Rural	2007 (25.4)	306 (17.2)	53.68 ***	40 (23.8)	40 (23.8)	0.00
	Urban	5882 (74.6)	1470 (82.8)		128 (76.2)	128 (76.2)	
Hypertension	Yes	4472 (56.7)	987 (55.5)	0.78	93 (55.4)	93 (55.4)	0.00
	No	3416 (43.3)	790 (44.5)		75 (44.6)	75 (44.6)	
Diabetes	Yes	1865 (23.6)	343 (19.3)	15.40 ***	149 (88.7)	149 (88.7)	0.00
	No	6024 (76.4)	1433 (80.7)		19 (11.3)	19 (11.3)	
Depressive symptoms	Yes	2761 (35.0)	379 (21.3)	123.64 ***	23 (13.7)	23 (13.7)	0.00
	No	5127 (65.0)	1398 (78.7)		145 (86.3)	145 (86.3)	
Visual difficulties	Yes	3318 (42.1)	510 (28.7)	107.88 ***	33 (19.6)	33 (19.6)	0.00
	No	4571 (57.9)	1266 (71.3)		135 (80.4)	135 (80.4)	
Hearing difficulties	Yes	2017 (25.6)	370 (20.8)	17.46 ***	8 (4.8)	8 (4.8)	0.00
	No	5872 (74.4)	1406 (79.2)		160 (95.2)	160 (95.2)	
Currently smoking	Yes	965 (12.2)	160 (9.0)	17.71 ***	3 (1.8)	3 (1.8)	0.00
	No	6923 (87.8)	1617 (91.0)		165 (98.2)	165 (98.2)	
Currently drinking	Yes	2116 (26.8)	563 (31.7)	17.10 ***	39 (23.2)	39 (23.2)	0.00
	No	5773 (73.2)	1214 (68.3)		129 (76.8)	129 (76.8)	
Involving in cognitive activities	Yes	6407 (81.2)	1611 (90.7)	92.28 ***	160 (95.2)	160 (95.2)	0.00
	No	1481 (18.8)	165 (9.3)		8 (5.8)	8 (5.8)	
Involving in physical Activities	Yes	2112 (26.8)	920 (51.8)	421.85 ***	43 (25.6)	43 (25.6)	0.00
	No	5777 (73.2)	856 (48.2)		125 (74.4)	125 (74.4)	
Contact with children	Yes	7785 (98.7)	1765 (99.3)	5.05 *	168 (100.0)	168 (100.0)	-
	No	104 (1.3)	12 (0.7)		0 (0.00)	0 (0.00)	
Contact with relatives	Yes	6824 (86.5)	1639 (92.3)	44.56 ***	164 (97.6)	164 (97.6)	0.00
	No	1065 (13.5)	137 (7.7)		4 (2.4)	4 (2.4)	
Contact with friends	Yes	7468 (94.7)	1731 (97.5)	24.81 ***	168 (100.0)	168 (100.0)	-
	No	421 (5.3)	45 (2.5)		0 (0.00)	0 (0.00)	
Currently working	Yes	2320 (29.4)	505 (28.4)	0.68	45 (26.8)	45 (26.8)	0.00
	No	5569 (70.6)	1272 (71.6)		123 (73.2)	123 (73.2)	
Engaging in lifelong education	Yes	851 (10.8)	499 (28.1)	361.35 ***	21 (12.5)	21 (12.5)	0.00
	No	7037 (89.2)	1277 (71.9)		14 7 (87.5)	14 7(87.5)	
Doing volunteer work	Yes	282 (3.6)	160 (9.0)	97.97 ***	1 (0.6)	1 (0.6)	0.00
	No	7607 (96.4)	1617 (91.0)		167 (99.4)	167 (99.4)	

Note. * *p* < 0.05, *** *p* < 0.001.

**Table 3:** Association between TV viewing and cognitive dysfunction.

Variables	Before Matching			After Matching		
	B (S.E.)	Wald	OR (95% CI)	B (S.E.)	Wald	OR (95% CI)
TV viewing Yes	0.26 (0.06)	16.43	1.27 (1.12–1.45) ***	0.66 (0.25)	6.80	1.94 (1.18–3.21) **
Age	0.02 (0.01)	39.02	1.02 (1.01–1.03) ***	−0.01 (0.03)	0.09	0.99 (0.93–1.05)
Sex Female	0.02 (0.05)	0.24	1.03 (0.92–1.15)	−0.30 (0.42)	0.51	0.73 (0.32–1.69)
Education	0.03 (0.01)	34.98	1.03 (1.02–1.04) ***	−0.04 (0.04)	1.13	0.95 (0.87–1.04)
Residential location Rural	0.39 (0.05)	54.35	1.49 (1.34–1.65) ***	0.63 (0.36)	3.04	1.88 (0.92–3.85)
Hypertension No	0.19 (0.04)	17.39	1.22 (1.11–1.33) ***	0.76 (0.30)	6.37	2.14 (1.18–3.88) *
Diabetes No	−0.14 (0.05)	7.31	0.86 (0.77–0.96) **	0.09(0.43)	0.05	1.10 (0.47–2.57)
Depressive symptoms Yes	0.26 (0.05)	26.87	1.31 (1.18–1.44) ***	0.08 (0.40)	0.04	1.08 (0.49–2.38)
Visual difficulties Yes	0.15 (0.04)	10.03	1.17 (1.06–1.28) **	0.03 (0.35)	0.01	1.03 (0.51–2.08)
Hearing difficulties Yes	0.29 (0.05)	29.22	1.33 (1.20–1.48) ***	0.69 (0.61)	1.26	1.99 (0.59–6.67)
Currently smoking Yes	−0.02 (0.07)	0.11	0.97 (0.84–1.12)	−20.69 (15831.95)	0.00	0.00 (0.00–)
Currently drinking Yes	0.01 (0.05)	0.03	1.01 (0.90–1.13)	0.66 (0.40)	2.65	1.94 (0.87–4.33)
Involving in cognitive activities No	0.18 (0.05)	9.32	1.20 (1.07–1.35) **	1.09 (0.57)	3.65	2.98 (0.97–9.13)
Involving in physical activities No	0.01 (0.05)	0.01	1.01 (0.91–1.12)	0.74 (0.35)	4.47	2.11 (1.05–4.22) *
Contact with children No	−0.17 (0.20)	0.67	0.83 (0.55–1.24)	-	-	-
Contact with relatives No	0.36 (0.06)	28.78	1.45 (1.27–1.66) ***	−0.18 (0.81)	0.05	0.83 (0.16–4.08)
Contact with friends No	0.38 (0.10)	14.61	1.53 (1.25–1.86) ***	-	-	-
Currently working No	0.07 (0.05)	2.11	1.08 (0.97–1.20)	0.48 (0.38)	1.59	1.61 (0.76–3.40)
Engaging in lifelong education No	0.46 (0.07)	37.31	1.59 (1.37–1.84) ***	−0.31 (0.43)	0.54	0.72 (0.31–1.69)
Doing volunteer work No	0.35 (0.12)	8.02	1.41 (1.10–1.81) ***	−0.18 (1.48)	0.01	0.82(0.04–15.24)

Note. OR = Odds Ratio; CI = Confidence Interval; S.E. = Standard Error. The column B refers to unstandardized regression weight; S.E. is how much B (the unstandardized regression weight) can vary, which is similar to a standard deviation to a mean; Wald is the χ^2^ test statistic for the individual predictor variable used to determine the *p* value. * *p* < 0.05, ** *p* < 0.01, *** *p* < 0.001.
